# CT-based radiomics nomogram for overall survival prediction in patients with cervical cancer treated with concurrent chemoradiotherapy

**DOI:** 10.3389/fonc.2023.1287121

**Published:** 2023-12-07

**Authors:** Chao Xu, Wen Liu, Qi Zhao, Lu Zhang, Minyue Yin, Juying Zhou, Jinzhou Zhu, Songbing Qin

**Affiliations:** ^1^ Department of Radiation Oncology, The First Affiliated Hospital of Soochow University, Suzhou, China; ^2^ Department of Gastroenterology, The First Affiliated Hospital of Soochow University, Suzhou, China

**Keywords:** cervical cancer, radiomic, deep learning, predictive model, overall survival

## Abstract

**Background and purpose:**

To establish and validate a hybrid radiomics model to predict overall survival in cervical cancer patients receiving concurrent chemoradiotherapy (CCRT).

**Methods:**

We retrospectively collected 367 cervical cancer patients receiving chemoradiotherapy from the First Affiliated Hospital of Soochow University in China and divided them into a training set and a test set in a ratio of 7:3. Handcrafted and deep learning (DL)-based radiomics features were extracted from the contrast-enhanced computed tomography (CT), and the two types of radiomics signatures were calculated based on the features selected using the least absolute shrinkage and selection operator (LASSO) Cox regression. A hybrid radiomics nomogram was constructed by integrating independent clinical risk factors, handcrafted radiomics signature, and DL-based radiomics signature in the training set and was validated in the test set.

**Results:**

The hybrid radiomics nomogram exhibited favorable performance in predicting overall survival, with areas under the receiver operating characteristic curve (AUCs) for 1, 3, and 5 years in the training set of 0.833, 0.777, and 0.871, respectively, and in the test set of 0.811, 0.713, and 0.730, respectively. Furthermore, the hybrid radiomics nomogram outperformed the single clinical model, handcrafted radiomics signature, and DL-based radiomics signature in both the training (C-index: 0.793) and test sets (C-index: 0.721). The calibration curves and decision curve analysis (DCA) indicated that our hybrid nomogram had good calibration and clinical benefits. Finally, our hybrid nomogram demonstrated value in stratifying patients into high- and low-risk groups (cutoff value: 5.6).

**Conclusion:**

A high-performance hybrid radiomics model based on pre-radiotherapy CT was established, presenting strengths in risk stratification.

## 1 Introduction

Cervical cancer, as one of the most common gynecologic malignancies, is the fourth leading cause of cancer-related death among women around the world, with over 300,000 deaths worldwide per year (1, 2). In developing countries, the incidence of cervical cancer was approximately 15.7/100,000, and the risk of death due to cervical cancer was nearly 0.9% (3). With the advancement in radiotherapy and chemotherapy, chemoradiotherapy is currently the first-line treatment option for locally advanced cervical cancer and was demonstrated to be associated with improved overall survival compared with radiotherapy alone (4, 5). However, the overall survival of cervical cancer seems to be without significant progress, with the 5-year overall survival (OS) rate of all the cases still below 66.7%, less than 30% in small cell carcinoma of the cervix (6–8). Precise prediction of clinical outcomes may help physicians provide individualized treatment to cervical cancer patients with different risks and deliver timely intervention in patients with a high risk of death.

The rapid development of artificial intelligence (AI) over the past decade has created growing excitement, and its application in medicine is currently a hot topic. Radiomics, as an effective and widely researched case of AI application in medicine, transforming medical images into diggable data by the high-throughput extraction of quantitative features, shows a promising application in cancer diagnosis, evaluation of treatment response, and prediction of survival outcomes (9–13). Several studies have proved the value of radiomics in predicting recurrence and metastasis in cervical cancer patients treated with concurrent chemoradiotherapy (CCRT) (14–16). However, limited studies have focused on the prediction of the final survival outcome. Furthermore, the handcrafted radiomics features are restricted to the current recognition of medical images and the reserved knowledge of operators (17).

Deep learning, as one of AI’s most powerful and typical algorithms, can automatically learn and extract features via multiple processing layers. Compared to traditional feature extraction, deep learning reduces manual preprocessing steps and can provide more deep-going features (18). Adding deep learning-based features can further improve the performance of predictive models (19, 20).

In our research, we aimed to develop and validate a comprehensively hybrid radiomics nomogram based on pre-radiotherapy contrast-enhanced CT by integrating independent clinical risk factors, handcrafted radiomics signature, and deep learning (DL)-based radiomics signature to predict the overall survival of cervical cancer patients treated with CCRT.

## 2 Materials and methods

### 2.1 Patients

**Figure 1:**
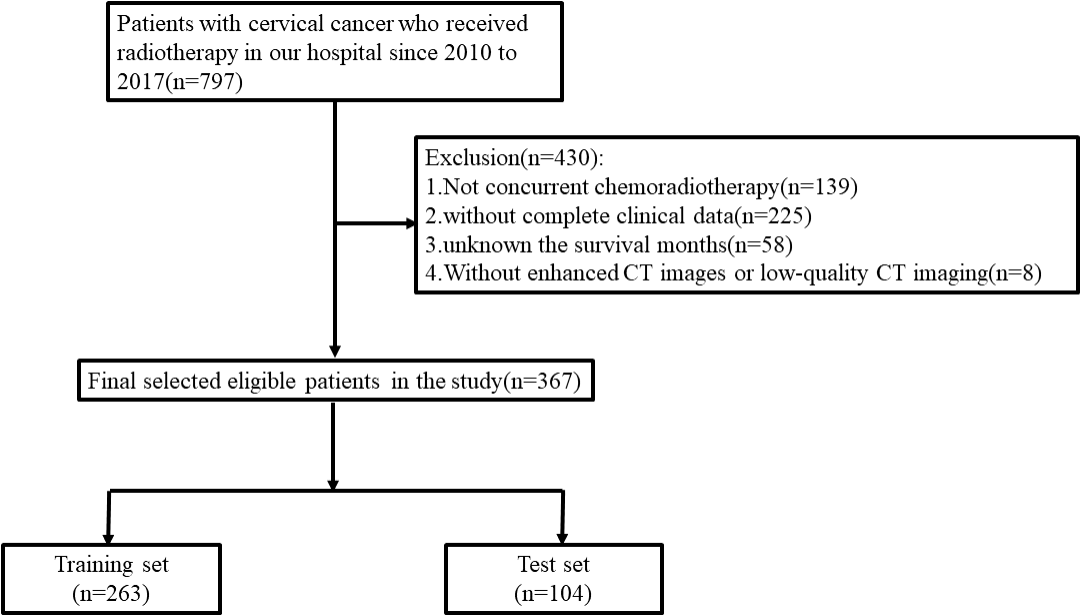
Flowchart of cervical cancer patients’ selection in the training set and test set.

The retrospective study was approved by the ethics committee of our institution. A total of 367 cervical cancer patients receiving CCRT between 2010 and 2017 in the First Affiliated Hospital of Soochow University were enrolled in our research. The training and test sets were split up at random from the enrolled patients at a ratio of 7:3 (**Figure 1**). The concrete inclusion and exclusion criteria are shown in **Supplementary Material**. Contrast-enhanced CT images were collected before radiotherapy. Baseline clinical-pathological data, including age, body mass index (BMI), pathological type, surgery, overall stage, and human papillomavirus (HPV) infection were obtained from the medical records. The tumor stage was strictly adherent to the 2018 International Federation of Gynecology and Obstetrics (FIGO) staging system.

### 2.2 Treatment and follow-up

All patients in this research received CCRT. For the radiotherapy regimen, patients underwent external radiotherapy (ERT) with or without intracavitary brachytherapy (IBT). ERT methods include three-dimensional conformal radiation therapy (3D-CRT) and intensity-modulated radiotherapy (IMRT), and CT simulation positioning was used. The total external radiation dose was DT45–50Gy, and the positive lymph node area in the pelvic and abdominal cavity can increase by 10–15 Gy. Intracavitary brachytherapy uses iridium-192 as the radiation source, with a single dose of 5–7 Gy; the dose at point A was 25–30 Gy. For the chemotherapy regimen, the choice depended on the decision of the professional multidisciplinary team and the tolerance of patients.

The last follow-up time was December 2019. Postoperative follow-up, including outpatient review, inpatient medical review, and telephone interview, was performed regularly. The follow-up information was recorded carefully in the hospital’s electronic patient record system. OS was defined as the date of pathological diagnosis to the date of death or of the last contact.

### 2.3 Imaging acquisition and tumor segmentation

**Figure 2:**
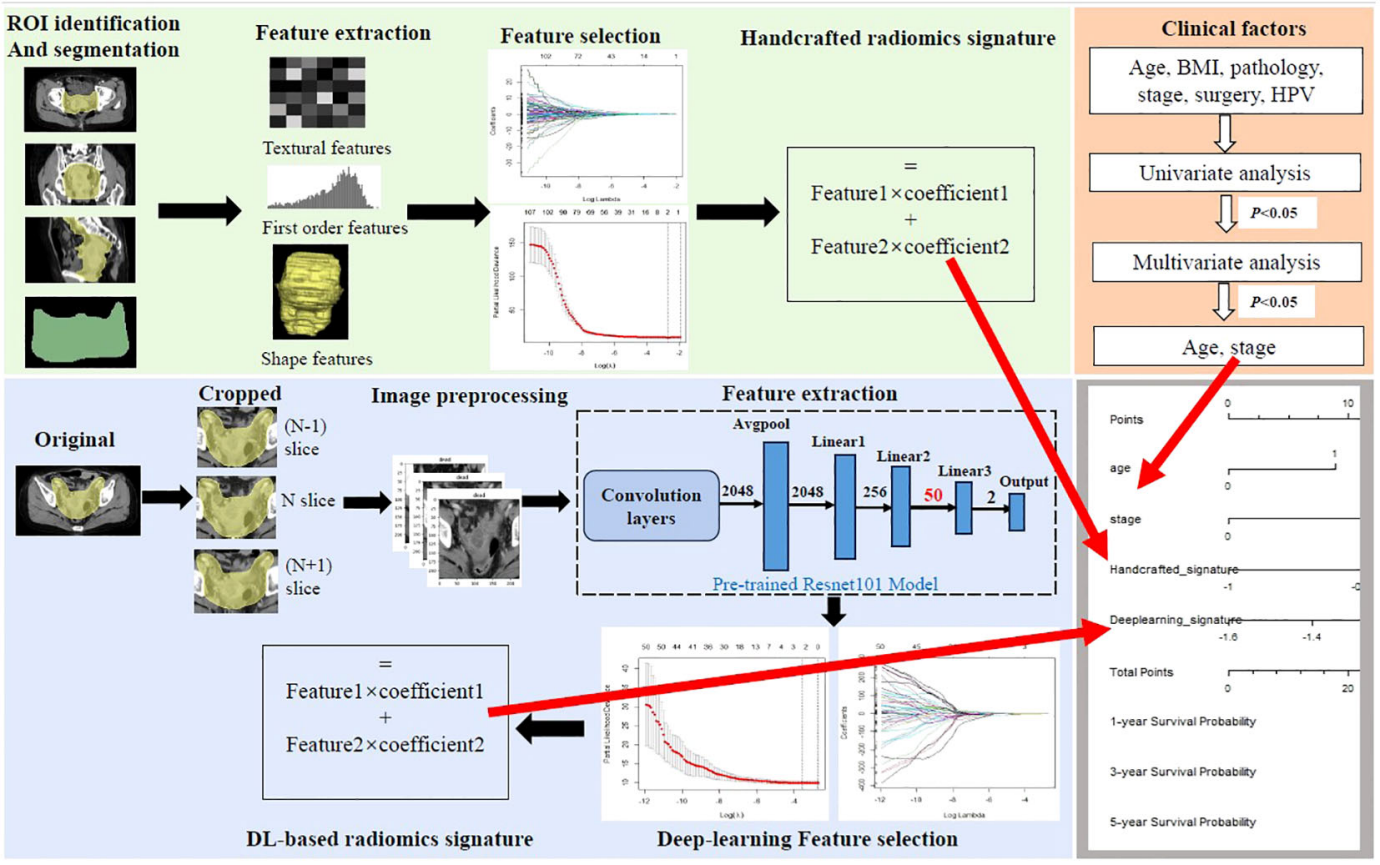
Workflow of the study. Handcrafted radiomics process including tumor segmentation, feature extraction, feature selection, and the construction of handcrafted radiomics signature. The deep learning (DL) radiomics process including image cropping, image preprocessing, feature extraction based on the pre-trained reconstructed ResNet101 model, feature selection, and the construction of DL-based radiomics signature. Univariate and multivariate analyses were performed to select independent clinical risk factors. The hybrid nomogram incorporating the handcrafted radiomics signature, the DL-based signature, and the independent clinical risk factors was constructed for clinical application.

All cervical cancer patients underwent a standard and systemic pelvic contrast-enhanced CT scan with 16-channel CT scanners (Philips Brilliance Big Bore CT, Philips, Bothell, WA, USA). Details regarding the CT scanning parameters were as follows: 120 kVp tube voltage, a tube current range of 250 to 350 mAs, 3 mm slice thickness, an image matrix of 512 × 512, a reconstruction slice thickness range of 1 or 2 mm, and standard B (body) reconstruction kernels. The patients’ CT data were saved in “DICOM” format from the image-archiving workstation in our hospital. The clinical target volume 1 (CTV1) was chosen as the region of interest (ROI). CTV1 was defined as the upper boundary (without para-abdominal aortic lymph node metastasis) for the bifurcation of the abdominal aorta, and the upper boundary (with para-abdominal aortic lymph node metastasis) should be extended appropriately, 7 mm around the blood vessels, inside the psoas major muscle and on the surface of the vertebral body. The lower boundary is 3 cm above the top of the vagina. The anterior boundary is the posterior wall of the bladder, and the posterior boundary is the mesorectum. The processing flow of image and clinical data is shown in **Figure 2**. Three radiologists who were blinded to the clinical-pathological data and had at least 5 years of work experience participated in the process of tumor segmentation. One radiologist manually delineated ROI slice-by-slice using the Treatment Planning System (TPS) equipped with four medical accelerators (cms, Monaco, pinnacle, and varian). The other two radiologists performed the role of reviewers and made corrections by consensus.

### 2.4 The handcrafted radiomics feature extraction/selection and radiomics signature building

A total of 107 originally handcrafted radiomics features were extracted using PyRadiomics (version 2.2.0), including 14 shape features, 18 intensity features, and 75 texture features. The Z-score normalization was applied to balance the distribution of feature intensity. Then, the least absolute shrinkage and selection operator (LASSO) Cox regression with 10-fold cross-validation was applied to select significant features with non-zero coefficients. The handcrafted radiomics signature was calculated as a linear combination of the selected features weighting by respective coefficients. The detailed process of feature extraction can be seen in **Supplementary Material**.

### 2.5 DL-based radiomics feature extraction/selection and radiomics signature building

Before the model pretraining and feature extraction, the largest ROI was selected as the center slice, 1 slice upward and 1 slice downward to represent each patient. These slices were saved in “jpg” format. Then, the selected image grayscale values were normalized to the range (−1, 1) using a min–max transformation, and the size of the cropped image was resized to 224 * 224 pixels.

The DL model was developed based on ResNet101 DL architecture (21). ResNet101 was first proposed by the teamwork of Kaiming He and was characterized by its strength in capturing the images’ finest details (22, 23). A dual pretrained ResNet101-based model was used to perform feature extractions. First, the base ResNet101 model (**Supplementary Material Figure S1A**) was pretrained on ImageNet (https://www.image-net.org). Then, the pretrained base ResNet101 model architecture and learned weights were retained to conduct the second pretraining in our own training sets, which is typically called transfer learning. The detailed process of the second pretraining was as follows. The last fully connected layer (FCL) and the classified layer (1,000 categories of ImageNet) of the base pretrained ResNet101 model were truncated and replaced with three linear layers and a new classifier layer (two categories of survival status) (**Supplementary Material Figure S1B**). Furthermore, in order to avoid overfitting and improve generalization, the methods of data augmentation were taken advantage of, such as flip, crop, rotation, and color, which were only applied in the training set. Finally, the dual pretraining weights of the new ResNet101 model were frozen and used as a feature extractor. A total of 50 deep learning features of each ROI image were output after the computation of the frontier convolutional layers, hidden layers, linear1, and linear2. The parameters of DL model development are described in **Supplementary Material**. The extracted DL features of three slices were averaged to represent each patient.

The same as the method of the construction of the handcrafted radiomics signature, the DL-based radiomics signature was also calculated as a linear combination of the selected features weighting by respective coefficients after LASSO Cox regression with 10-fold cross-validation.

### 2.6 Clinical model and development of hybrid radiomics nomogram

To explore the effect of clinical variables on the prognosis of cervical cancer patients and demonstrate the prognostic value of our hybrid model, the Cox regression analysis was applied to develop the clinical model. The Cox regression model was based on the results of multivariate analysis, in which the variables were first selected by the univariable regression analysis. The criterion for variable inclusion in univariable and multivariate analyses was p < 0.05.

The hybrid model was established by integrating the handcrafted radiomics signature, the DL-based radiomics signature, and the independent clinical risk factors into the multivariable Cox proportional hazards. We used the method of the scaled Schoenfeld residual test to check the proportional hazards (PH) assumption. The prognostic value of the hybrid model was compared with the single clinical model, the handcrafted radiomics signature, and the DL-based radiomics signature in both the training set and test set using C-index and areas under the receiver operating characteristic (ROC) curve (AUCs). A hybrid radiomics nomogram was built to provide an individual evaluation of the overall survival. The Brier scores and calibration curves were used to evaluate the fit degree of the actual outcome and the models’ predictions in all sets. Decision curve analysis (DCA) was plotted to quantify the net benefits to a range of threshold probabilities in test sets, demonstrating clinical usefulness. Furthermore, the risk score of each patient was computed based on the established hybrid radiomics model, and patients were stratified into high- and low-risk groups according to their risk scores using X-tile software (version 3.6.1). The Kaplan–Meier survival analysis was applied to calculate the survival rates between the two risk groups and compared using the log-rank test.

### 2.7 Statistical analysis

All statistical analyses were performed in R software (version 4.1.0) (http://www.R-project.org). The detailed R packages applied in the analysis can be seen in **Supplementary Material**. Continuous variables were compared using the Wilcoxon test, and categorical variables were compared using a chi-square test or Fisher’s exact test. A two-sided p-value of <0.05 was considered statistically significant.

## 3 Results

### 3.1 The characteristics of patients

A total of 367 cervical cancer patients who received CCRT were enrolled in the research: 263 in the training set and 104 in the test set. The detailed clinical-pathological characteristics are summarized in **Table 1**. There were no significant differences between the training set and test set in terms of age, BMI, pathological type, surgery, overall stage, and HPV infection. The median follow-up period in the training and test sets were 56 and 53 months, respectively. A total of 46 (17.5%) patients and 20 (19.2%) patients were confirmed dead in the training and test sets during the follow-up period.

**Table 1:** Baseline clinical characteristics of cervical cancer patients receiving CCRT in the training and test sets.

Characteristics	Training set (n = 263)	Validation set (n = 104)	p
Age (%)			0.325
<60	198 (75.3)	84 (80.8)	
≥60	65 (24.7)	20 (19.2)	
BMI (%)			0.746
<24	168 (63.9)	69 (66.3)	
≥24	95 (36.1)	35 (33.7)	
Pathology (%)			0.977
Squamous carcinoma	244 (92.8)	96 (92.3)	
Adenocarcinoma	17 (6.5)	7 (6.7)	
Adenosquamous carcinoma	2 (0.8)	1 (1.0)	
Surgery (%)			0.959
No	86 (32.7)	35 (33.7)	
Yes	177 (67.3)	69 (66.3)	
Overall stage (FIGO) (%)			0.864
I/II	224 (85.2)	90 (86.5)	
III	39 (14.8)	14 (13.5)	
HPV infection			0.682
No	32 (12.2)	15 (14.4)	
Yes	231 (87.8)	89 (85.6)	
Status			0.810
Alive	217 (82.5)	84 (80.8)	
Dead	46 (17.5)	20 (19.2)	
Follow-up time (months) Median (range)	56 (3,110)	53 (3,109)	0.662

CCRT, concurrent chemoradiotherapy; BMI, body mass index; FIGO, International Federation of Gynecology and Obstetrics; HPV, human papillomavirus.

### 3.2 Feature selection and signature development

A total of 107 originally handcrafted radiomics features and 50 DL-based features for each patient were extracted. Then, two handcrafted radiomics features and two DL-based radiomics features that were most useful to predict OS were selected using LASSO in the training set to build a radiomics signature (**Supplementary Material Table S3**). **Supplementary Material Figure S2** depicts the procedures of variable selection. Then, the two types of radiomics signatures were calculated according to the method mentioned above.

**Figure 3:**
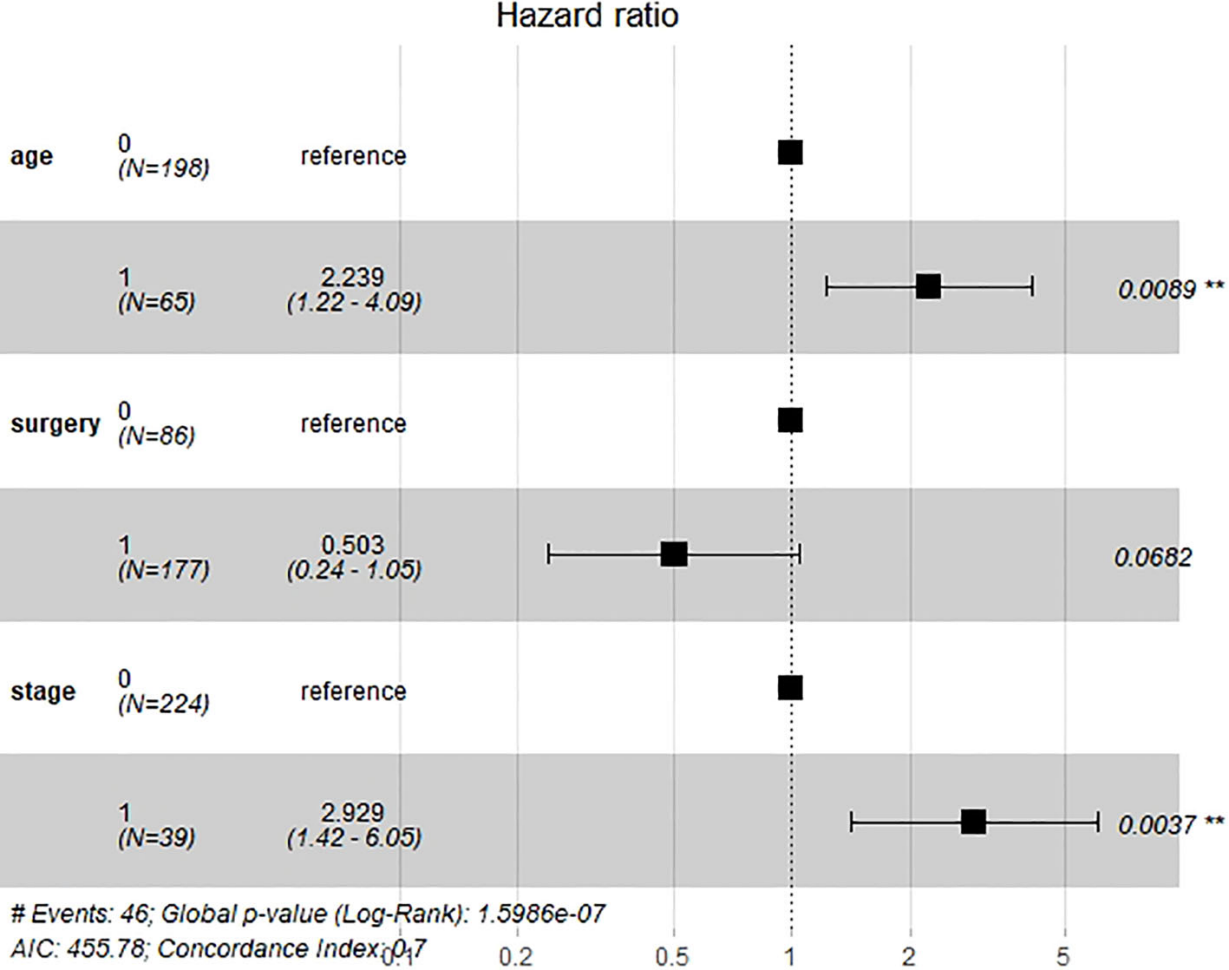
Forest plot of multivariate Cox regression analysis of overall survival (OS).

Six important clinical variables mentioned above were admitted into univariable and multivariate Cox regression analyses. Age and stage were demonstrated as independent clinical risk factors and were used to build the clinical model (**Figure 3**). The Kaplan–Meier survival analysis indicated that patients with a higher FIGO stage had a lower OS (**Supplementary Material Figure S3**).

### 3.3 The performance of handcrafted radiomics signature, DL-based radiomics signature, clinical model, and hybrid radiomics nomogram

The handcrafted radiomics signature yielded a C-index of 0.628, and the AUCs for 1, 3, and 5 years were 0.818, 0.587, and 0.676, respectively, in the test set. The DL-based radiomics signature yielded a C-index of 0.652, and the AUCs for 1, 3, and 5 years were 0.485, 0.659, and 0.648, respectively, in the test set. The clinical model yielded a C-index of 0.663, and the AUCs for 1, 3, and 5 years were 0.696, 0.699, and 0.629, respectively, in the test set.

**Figure 4:**
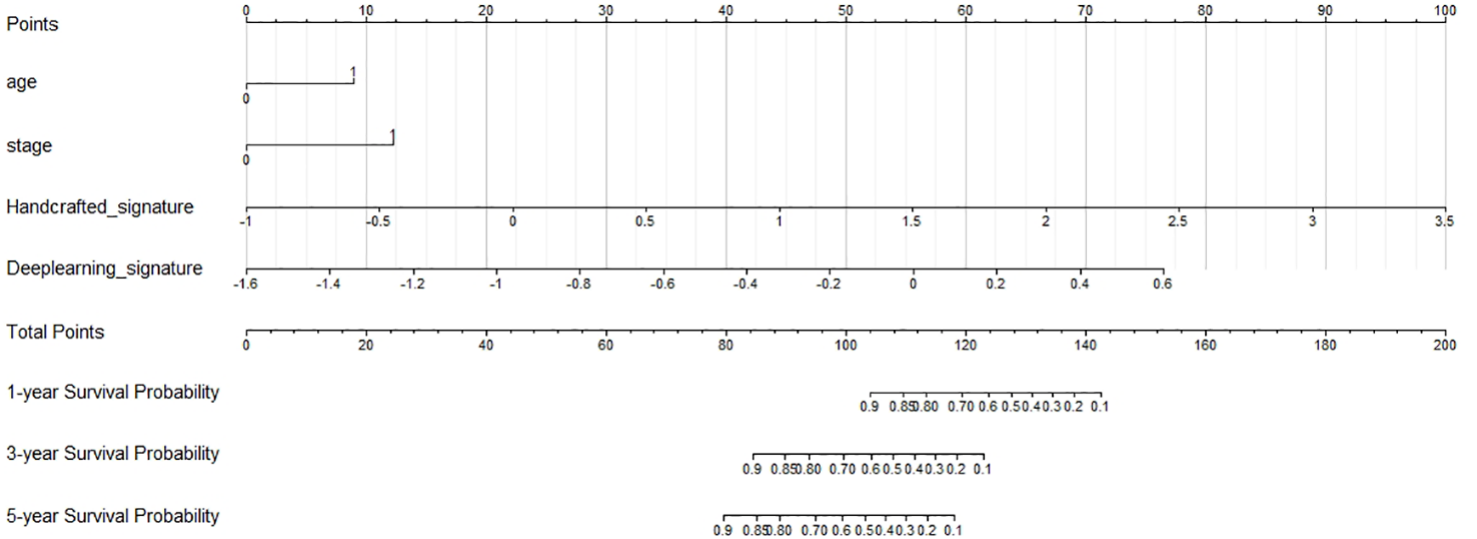
Nomogram predicting the overall survival (OS) rates for 1, 3, and 5 years of patients with cervical cancer treated with concurrent chemoradiotherapy.

**Figure 5:**
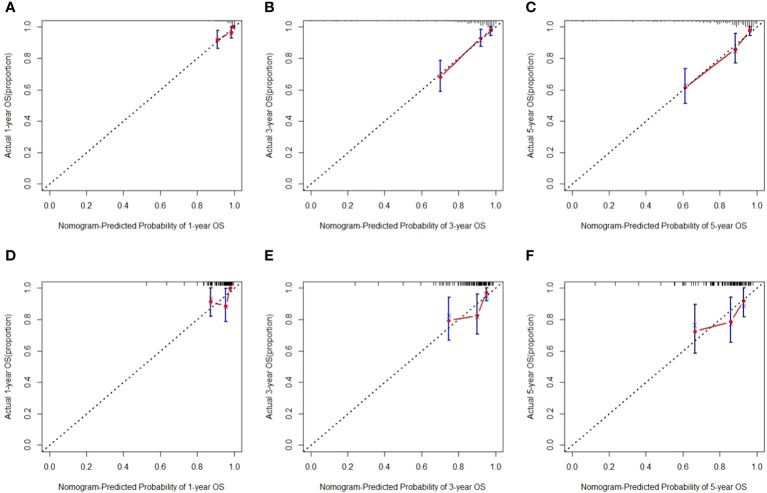
Calibration curves for predicting overall survival (OS) in the training **(A–C)** and test sets **(D–F)**.

**Figure 6:**
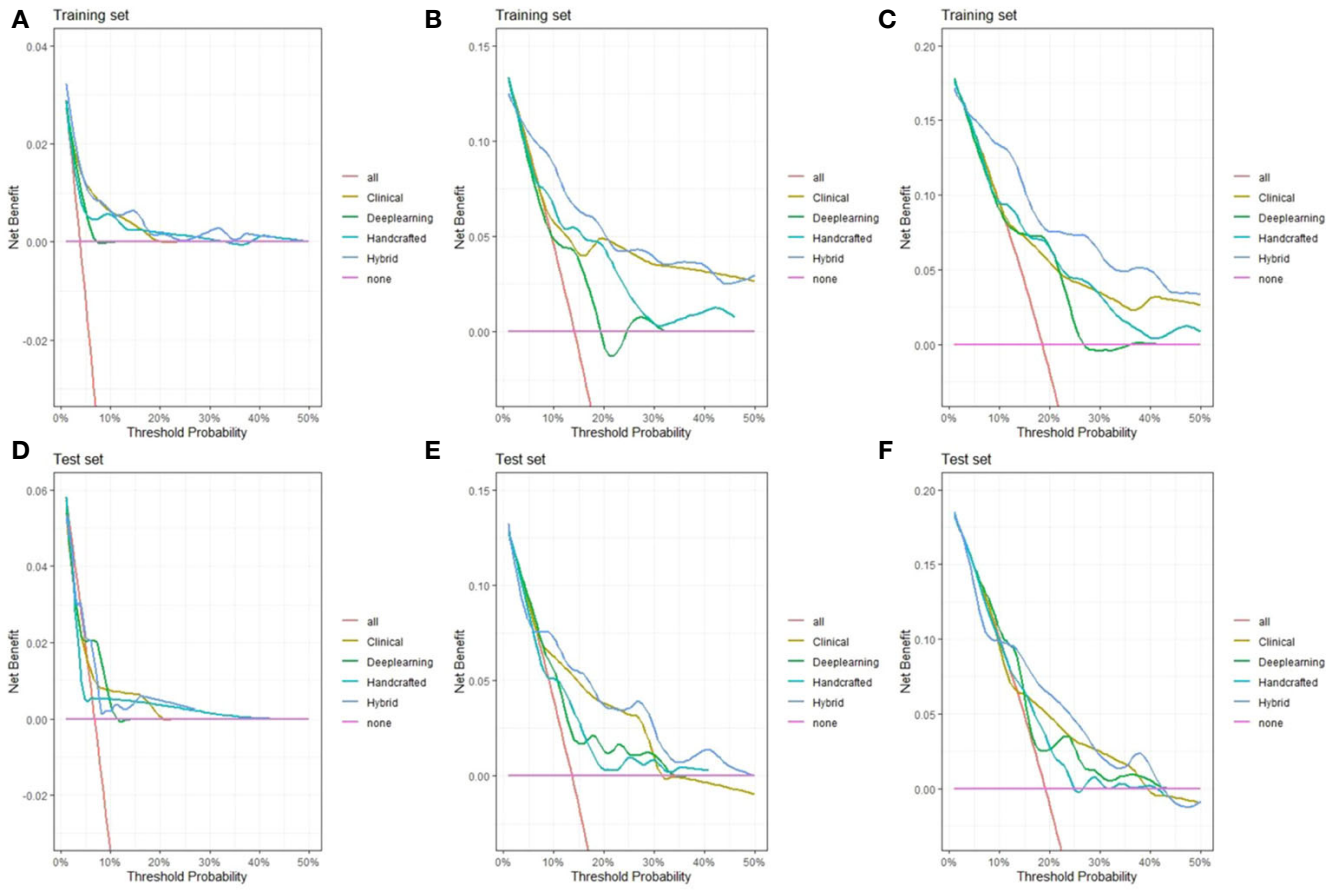
Decision curve analysis of the hybrid nomogram and the single clinical model, handcrafted radiomics signature, and DL-based radiomics signature for 1-year **(A)**, 3-year **(B)**, and 5-year **(C)** overall survival (OS) in the training set **(A–C)** and test set **(D–F)**.

We then created a hybrid radiomics nomogram by integrating handcrafted radiomics signature, DL-based radiomics signature, age, and FIGO stage into the multivariable Cox proportional hazards model (**Figure 4**). The chi-square test of the Schoenfeld residuals demonstrated that the hybrid Cox model satisfied the PH assumption (p > 0.05) (**Supplementary Material Figure S4**). The hybrid radiomics model achieved a C-index of 0.793 in the training set and 0.721 in the test set. The AUCs for 1, 3, and 5 years in the training set were 0.833, 0.777, and 0.871, respectively. The AUCs for 1, 3, and 5 years in the test set were 0.811, 0.713, and 0.730, respectively. Both C-index and AUCs confirmed that the hybrid radiomics nomogram outperformed the single signatures in the training and test sets. We summarized the concrete results in **Table 2**. The Brier scores (all <0.25) and the calibration curves presented that nomogram-predicted outcomes had good agreement with the actual survival (**Figures 5A–F**). In addition, the DCA curves in **Figure 6** showed that the hybrid nomogram for predicting 3-year and 5-year OS had greater net benefits compared with the single signatures in the training and test sets, indicating that the hybrid nomogram had better clinical practice.

**Table 2:** Predictive performances of clinical model, handcrafted radiomics signature, DL-based radiomics signature, and hybrid nomogram.

Model		AUC 1-year	AUC 3-year	AUC 5-year	Brier score 1-year	Brier score 3-year	Brier score 5-year	C-index
Clinical	Training	0.774	0.693	0.690	0.035	0.099	0.126	0.671
	Test	0.696	0.699	0.629	0.062	0.111	0.153	0.663
Handcrafted	Training	0.697	0.703	0.825	0.036	0.107	0.126	0.722
	Test	0.818	0.587	0.676	0.065	0.114	0.143	0.628
Deep learning	Training	0.744	0.600	0.697	0.036	0.120	0.143	0.643
	Test	0.485	0.659	0.648	0.062	0.113	0.150	0.652
Hybrid	Training	0.833	0.777	0.871	0.033	0.089	0.103	0.793
	Test	0.811	0.713	0.730	0.070	0.111	0.154	0.721

DL, deep learning; AUC, area under the receiver operating characteristic curve.

### 3.4 The RSF risk stratification of patients

**Figure 7:**
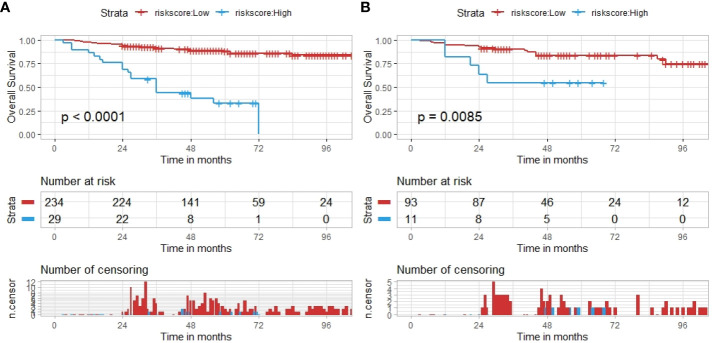
The hybrid nomogram risk stratification patients. **(A)** The nomogram risk stratification of patients in the training set. **(B)** The nomogram risk stratification of patients in the test set.

We calculated the risk score of each patient based on our hybrid nomogram and divided them into a high-risk group and a low-risk group. The optimal cutoff value for the risk score was 5.6 using X-tile (**Supplementary Material Figure S5**). The results of the Kaplan–Meier survival analysis and log-rank test indicated that the OS rates of the high-risk and low-risk groups were significantly different in both the training and test sets (**Figure 7**). The 2-, 3-, and 5-year OS rates of the two groups in the training sets were observed to be 96% *vs.* 76%, 69% *vs.* 55%, and 37% *vs.* 17%, respectively. The 2-, 3-, and 5-year OS rates of the two groups in the test sets were observed to be 94% *vs.* 73%,61% *vs.* 55%, and 34% *vs.* 18%, respectively.

## 4 Discussion

In this retrospective study, we established and validated a hybrid CT-based radiomics nomogram to predict overall survival in cervical cancer patients receiving CCRT. The hybrid radiomics nomogram developed by integrating handcrafted radiomics signature, DL-based radiomics signature, and independent clinical risk factors outperformed the model that used a single predictor. Furthermore, the risk scores calculated by the hybrid model could stratify cervical cancer patients into high- and low-risk groups with different prognoses, showing potential in clinical practice.

In recent years, there have been an increasing number of studies attempting to combine clinical data with imaging features to predict lymph node metastasis, treatment response, and prognosis (24–27). The imaging features contained the categories of radiomics (CT, MRI, and PET-CT) and pathological images. Zhang et al. (24) retrospectively analyzed the pre-treatment MRI images of 277 cervical cancer patients who received neoadjuvant chemotherapy (NACT) to predict tumor response to NACT in cervical cancer patients (the AUCs in the training set, internal validation set, and external validation set were 0.963, 0.940, and 0.910, respectively). Zhang et al. (25) developed a LASSO–Cox model to predict 5-year OS based on H&E-stained pathological images (the AUCs of the combined model, the single clinical model, and the pathological model in the test set were 0.750, 0.729, and 0.793, respectively). Chen et al. (26) developed a support vector machine (SVM) model to predict lymph node metastasis based on pre-therapy CT radiomics features (AUCs were 0.841 ± 0.035). On the one hand, our study aimed to establish a CT-based radiomics nomogram for overall survival prediction in patients with cervical cancer treated with CCRT, which is less researched by others. On the other hand, compared with the above studies, our research had a larger sample size and made full use of the CT radiomics data. Extracted features included handcrafted radiomics features and deep learning-based radiomics features. Our research supplemented the evidence of radiomics in the prediction of the prognosis of cervical cancer patients treated with CCRT.

Both radiomics and clinical-pathological data contained important prognostic information. Our research identified that age and FIGO stage were independent clinical risk factors of cervical cancer receiving CCRT, in line with other studies (28–30). In contrast to models constructed by a single type of clinical risk factors, the hybrid model combining clinical data with radiomics contained more prognostic information and had better predictive ability. Zhang et al. (13) developed a CT-based hybrid radiomics nomogram for the prediction of local recurrence-free survival in esophageal squamous cell cancer patients. A model incorporating handcrafted radiomics features and clinical features outperformed the single clinical model (C-index in the external validation set was 0.66 *vs.* 0.60), and adding deep learning features can further improve the accuracy of model prediction (C-index in the external validation set was 0.76). In our research, a hybrid nomogram established by integrating the handcrafted radiomics signature, the DL-based radiomics signature, and the independent clinical risk factors outperformed a single predictor (C-index in test set: 0.721).

In contrast with the traditional medical image assessments that mainly focused on qualitative features like tumor density, regularity of tumor margins, tumor enhancement pattern, and anatomical relationship with surrounding tissues, radiomics analysis could output many high-throughput quantitative features, enabling more objective analysis and evaluation of medical images (11). Furthermore, radiomics had strength in overcoming the spatial and temporal specificity of the whole cancer course, compared with genomics and proteomics (31). Multiple studies have demonstrated that radiomics could reflect the heterogeneity of tumor cells and tumor microenvironments (32–34). In our research, we extracted 107 handcrafted radiomics features. We finally selected one of the gray-level co-occurrence matrix (GLCM) features (Joint Average) and one of the gray-level dependence matrix (GLDM) features (Gray-Level Non-Uniformity) with LASSO regression to calculate the handcrafted radiomics signature. GLCM is a method of second-order statistical texture analysis that provides more information about texture by considering relationships between the intensity of pairs of neighboring pixels/voxels. GLCM can map the relationship between voxels within the ROI (35). Joint Average, as one of the GLCM features, can return the mean gray-level intensity of the distribution. GLDM features are computed based on the overall spatial relations of intensities, which describe the relations between intensities of every central pixel and all its neighbors within a window (36). Gray-Level Non-Uniformity, as one of the GLDM features, can return the similarity of gray-level intensity values in the images.

Deep learning is characterized by automatically learning from a huge amount of image data and extracting features via convolutional operations. Conventional radiomics needs handcrafted drawing ROI, which relies on clinicians’ professional knowledge, but it is time-consuming. In contrast, deep learning can simplify the multi-step process by directly inputting a large dataset of labeled images with greater reproducibility (37). In addition, deep learning can dig out in-depth information hiding in images via multiple processing layers, complementing radiomics further (38). In this research, we chose a dual pretrained ResNet101-based model to perform feature extractions. On the one hand, the ResNet101 model is capable of training up to 100 layers of deep networks. On the other hand, the ResNet101 model can better tackle the problem of gradient vanishing by introducing the residual layers, guaranteeing model accuracy (39). The ResNet101 model has been demonstrated to present strength in the identification of medical images compared with other convolutional neural networks (CNNs) (12). Considering that deep learning requires a large amount of image data to avoid overfitting and improve the adaptability of the learned model, we first pretrained the base ResNet101 model on the ImageNet database. Then, we applied data augmentation methods, such as flipping, cropping, rotation, and coloring, to conduct the second pretraining in the newly developed ResNet101 model (mentioned above).

Although our research presented some favorable results, there were still some limitations that needed to be raised. First, this research was retrospective and single-center. The number of enrolled patients was limited in the training and test sets. Multi-center and prospective studies were expected to validate the constructed hybrid nomogram further. Second, the hybrid nomogram is not a fully automated model, as it requires specialists to undergo manual ROI annotation on the pre-radiotherapy CT. This process inevitably had some bias influencing the final radiomics features extraction. Third, this research constructed a model only based on the pre-radiotherapy CT. More prognostic information about cervical cancer can be explored if combined with other types of medical images, such as pathological images, MRI images, and PET/CT images. Fourth, our research applied traditional Cox proportional hazards (CoxPH) analysis to establish a CT-based radiomics nomogram and did not attempt to compare with the machine learning (ML) methods, such as random survival forest (RSF) and deep learning (Deepsurv), which are currently a hot topic. ML did not need to assume that the influence of all variables on the risk function is linear and presented strengths in tackling larger sample sizes and high-dimensional data compared with the traditional statistical methods (40). Our research needed further improvement in the future.

## 5 Conclusions

In conclusion, we developed and validated a CT-based hybrid radiomics nomogram that integrated independent clinical risk factors, and handcrafted and DL-based radiomics signature to predict overall survival in cervical cancer patients treated with concurrent chemoradiotherapy. The hybrid radiomics nomogram exhibited favorable performance for predicting prognosis and had the potential for guiding individualized treatment.

## Data availability statement

The raw data supporting the conclusions of this article will be made available by the authors, without undue reservation.

## Ethics statement

The studies involving humans were approved by the Ethics Committee of the First Affiliated Hospital of Soochow University. The studies were conducted in accordance with the local legislation and institutional requirements. The participants provided their written informed consent to participate in this study.

## Author contributions

CX: Conceptualization, Data curation, Writing – original draft. WL: Formal Analysis, Methodology, Writing – original draft, Conceptualization. QZ: Data curation, Writing – original draft. LZ: Data curation, Writing – original draft. MY: Methodology, Writing – original draft. JYZ: Writing – review & editing. JZZ: Methodology, Writing – review & editing. SQ: Writing – review & editing, Data curation.
