# Supplementary material for: CT-based radiomics nomogram for overall survival prediction in patients with cervical cancer treated with concurrent chemoradiotherapy
Source: Front Oncol. 2023 Dec 7;13:1287121. doi: 10.3389/fonc.2023.1287121 (PMC10755472; doi:10.3389/fonc.2023.1287121)
Supplement: Supplementary file 1 [file DataSheet_1.docx]

**Supplementary Materials**

**The inclusion/exclusion criteria of participants.**

The inclusion criteria were: (1) pathologically diagnosed as cervical cancer; (2) older than 18 years old; (3) perform positioning CT scan and radiotherapy in the Department of Radiotherapy; (4) No other malignant tumors or serious heart and lung diseases. Exclusion criteria: (1) patients not treated with concurrent chemoradiotherapy; (2) patients without complete clinical data; (3) patients without follow-up information; (4) patients without enhanced CT images or low-quality CT imaging.

**The details of handcrafted radiomics feature extraction.**

Features extracted in this study included three groups: (I) 18 intensity statistics features; (II) 14 shape-based features; (III) 75 textural features consisting of 24 gray level co-occurrence texture matrices (GLCM) features, 14 gray level dependence matrix (GLDM) features, 16 gray level run-length texture matrices (GLRLM) features, 16 gray level size zone matrix (GLSZM) features and 5 neighborhood gray tone difference matrix (NGTDM) features. The specific hyperparameters were listed in **Table S1**. The detailed mathematical definitions and illustrations of these radiomic features can be found within the Pyradiomics documentation (https://pyradiomics.readthedocs.io).

**The detailed parameters of DL model development**

The initial learning rate is 0.001. The first 7 epochs are the initial learning rate, and it multiplies to 0.1 every 7 epochs. We retained the model and corresponding weights with high accuracy when the training stopped. Other parameters of the network were listed in **Table S2**. We used the following loss function:

*nn.CrossEntropyLoss : loss*(*x*,*class*)​=−*log*(∑*j*​*exj*​*ex*[*class*]​​)=−*x*[*class*]​+*log*(*j*∑​*exj*​)​

**The detailed R packages used of statistical analysis**

The “glmnet” package was used to perform the LASSO Cox regression model analysis. The “survival” package was used for Kaplan–Meier survival analysis. The “survminer” package was used for plotting Kaplan–Meier survival curves and performing the log-rank test. The “rms” package was used for Cox proportional hazards regression, nomograms, and calibration curves. The “dcurves” package was used for plotting DCA curves.

**Table S1**. The parameter settings of radiomic feature extraction in Pyradiomics.

| **Setting item** | **value** |
| --- | --- |
| normalize | True |
| normalize scale | 100 |
| resampled pixel spacing | (1, 1, 1) |
| other parameters | default |

**Table S2**. The parameter settings of DL model development.

| **Setting item** | **value** |
| --- | --- |
| learning rate | 0.001 |
| optimize | Adam |
| loss function | nn.CrossEntropyLoss |
| epochs | 10 |
| batch size | 16 |

**Table S3.** **Handcrafted and DL-based radiomics features associated with OS selected by LASSO COX analysis.**

| **Handcrafted radiomics features** | **coefficient** |
| --- | --- |
| original_glcm_JointEnergy | 0.0979 |
| original_gldm_GrayLevelNonUniformity | 0.4129 |
| **DL-based radiomics features** | **coefficient** |
| Feature2 | -0.1346 |
| Feature45 | -0.1325 |


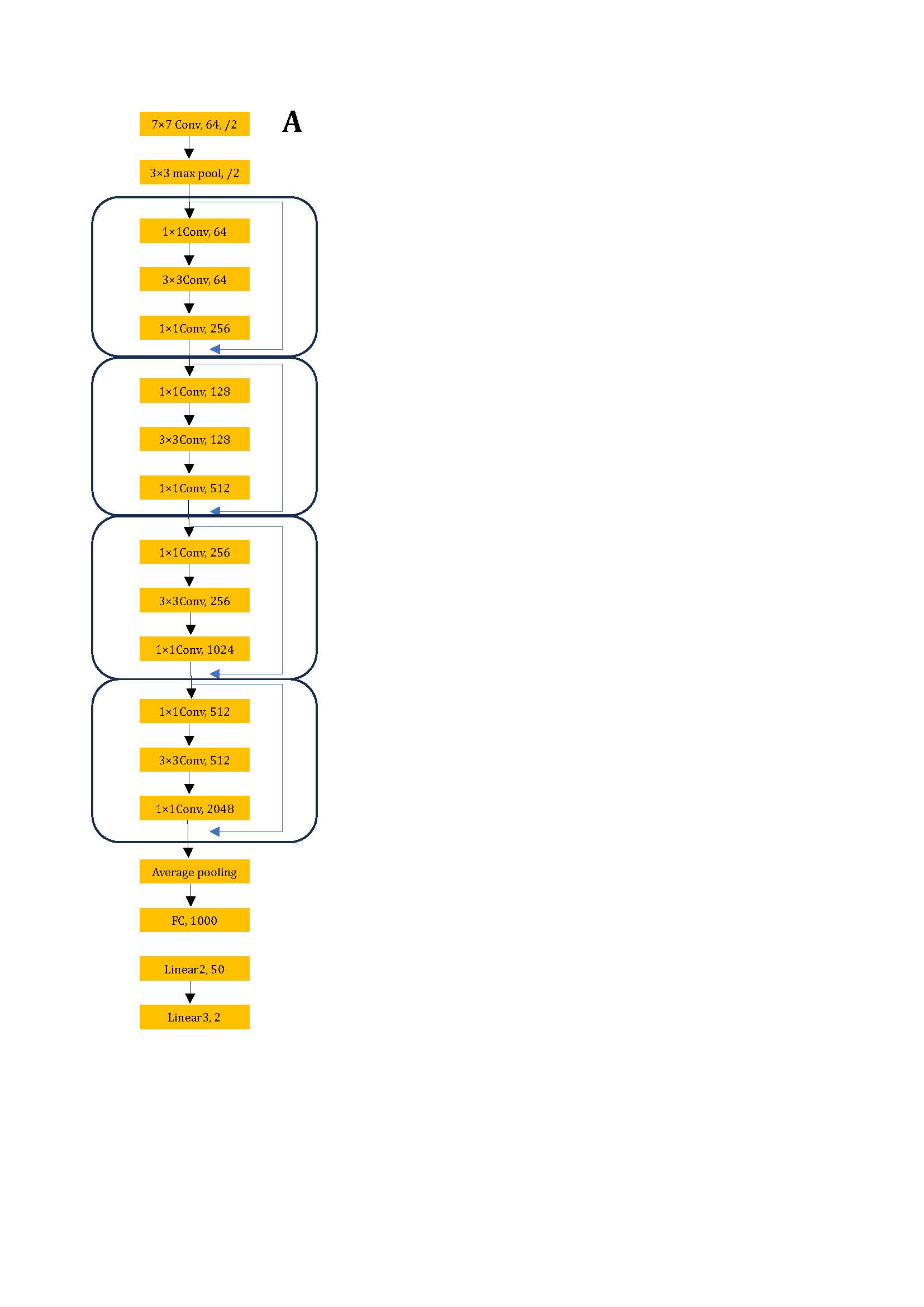


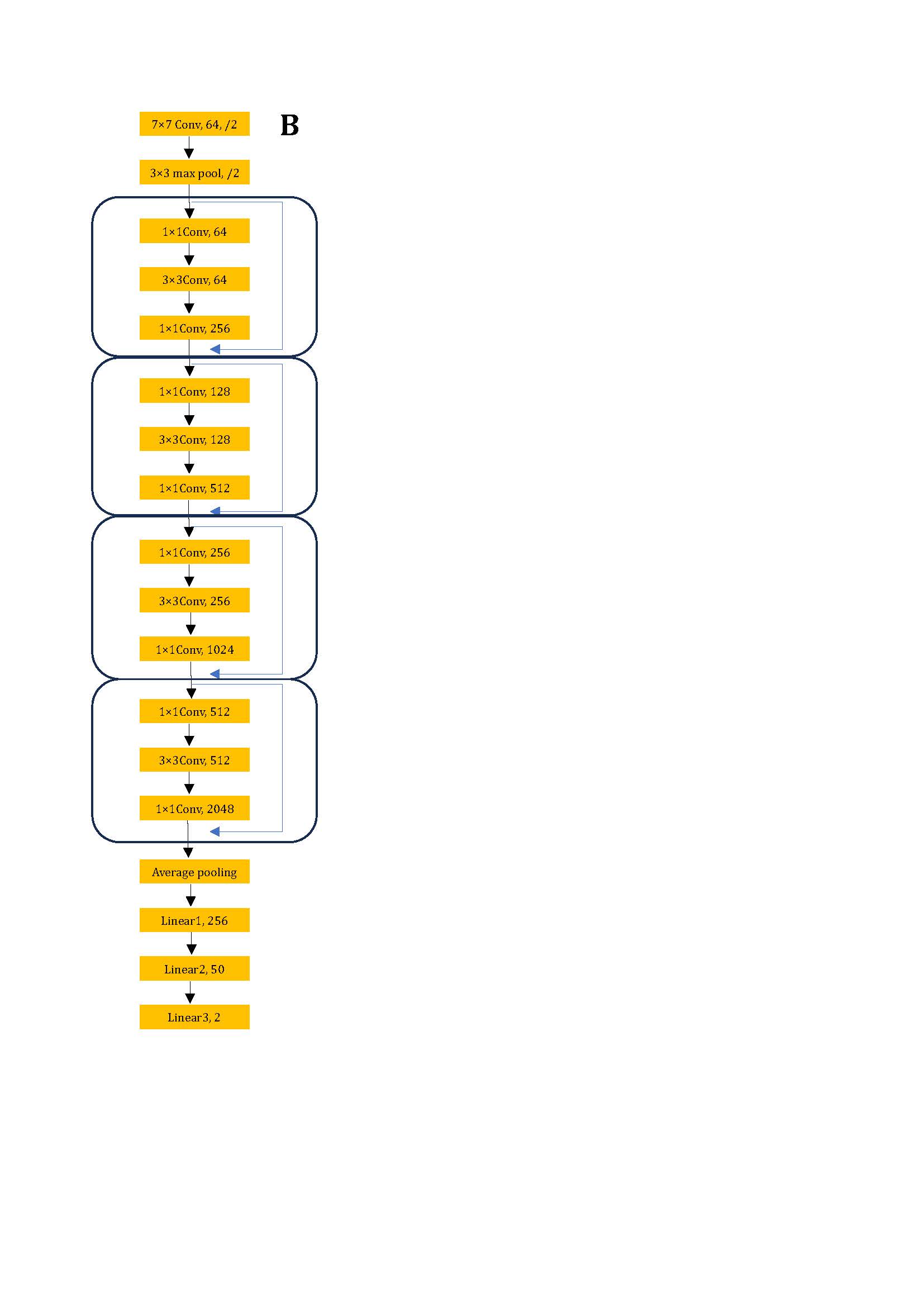


**Figure S1. (A)The base Resnet 101 model (B)The new-constructed Resnet 101 model (truncate the last FC in the base Resnet 101 model and replace it with three linear layers)**


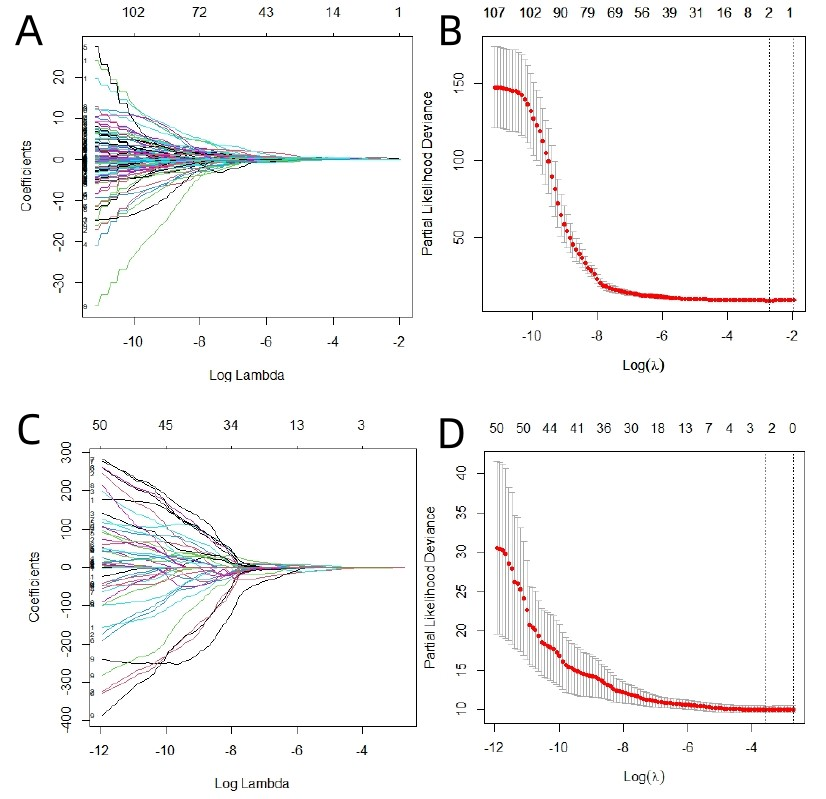


**Figure S2. The results of LASSO regression analysis for** **Handcrafted and DL-based radiomics features. (A) LASSO coefficient profiles of the expression of 107 Handcrafted radiomics features. (B) Selection of the λ in the LASSO regression analysis via 10-fold cross-validation for Handcrafted radiomics features. (C) LASSO coefficient profiles of the expression of 50 DL-based radiomics features. (D) Selection of the λ in the LASSO regression analysis via 10-fold cross-validation for DL-based radiomics features. The dashed line represents the optimal value under the minimum standard (right) and the "one standard error" standard (left).**


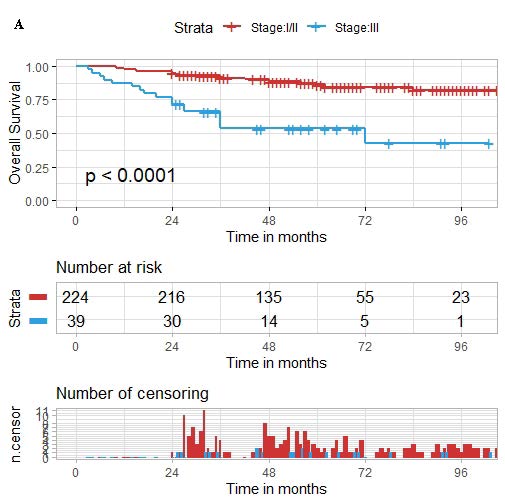

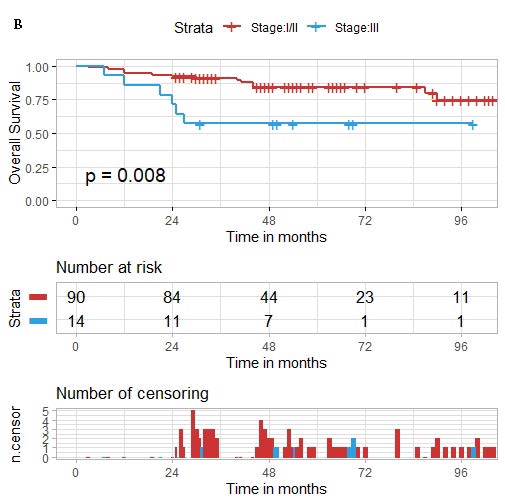


**Figure S3. OS survival curves of patients in FIG0 low-stage and high-stage groups in (A) training, (B) test sets. Significant differences were observed in the two sets (log-rank test p<0.05).**


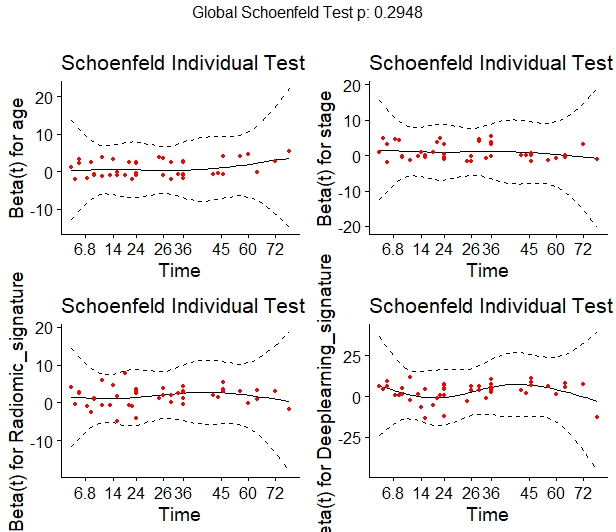


**Figure S4. The presentation of the Schoenfeld Residuals Test**


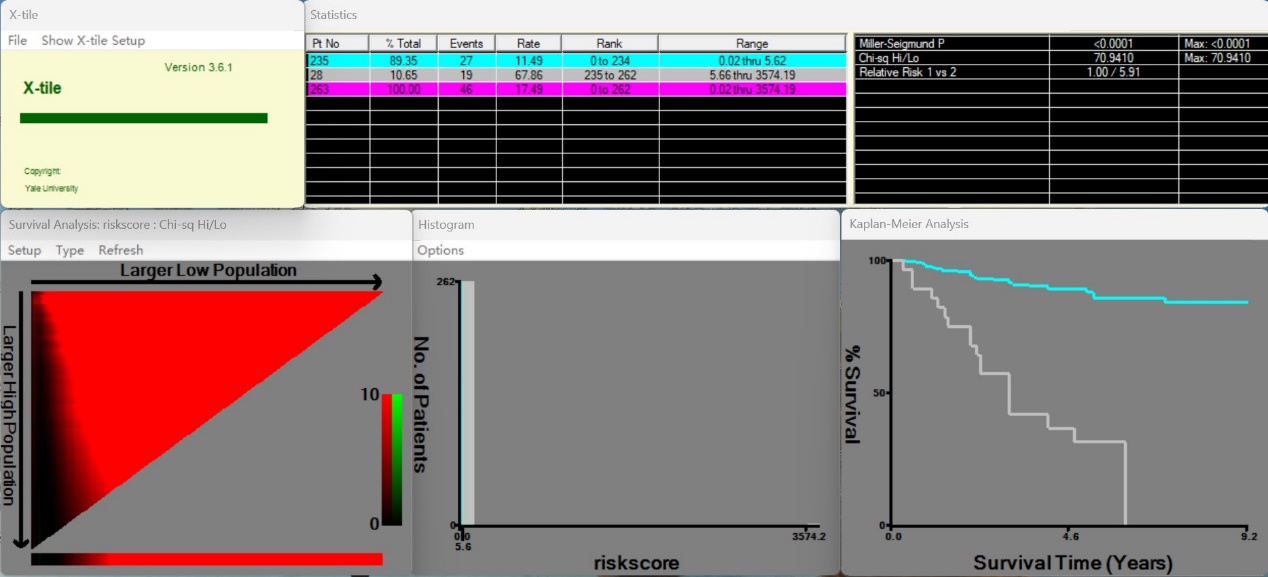


**Figure S5. Selecting the optimal cut-off value of risk scores via the X-tile tool.**
